# #Brachytherapy: Physicians As Influencers on Instagram

**DOI:** 10.7759/cureus.22524

**Published:** 2022-02-23

**Authors:** Anam Z Kesaria, Milan Bimali, Mausam Patel, Arpan Prabhu, Sarah Kesaria, Fen Xia

**Affiliations:** 1 Radiation Oncology, University of Arkansas for Medical Sciences, Little Rock, USA; 2 Statistics, University of Arkansas for Medical Sciences, Little Rock, USA; 3 Radiation Oncology, University of Texas Medical Branch at Galveston, Galveston, USA; 4 Radiation Oncology, Winthrop P. Rockefeller Cancer Institute, University of Arkansas for Medical Sciences, Little Rock, USA

**Keywords:** social media, radiation oncology, cervical cancer, brachytherapy, instagram

## Abstract

Purpose

We aimed to evaluate brachytherapy-related posts on Instagram by identifying patient concerns, the content of related posts, and user outreach.

Methods and materials

A list of top posts from searching #brachytherapy on May 7, 2021, were generated on a mobile device and all data are representative as of May 7, 2021. Searching for #brachytherapy resulted in 1010 posts which were analyzed using Instagram. The content was categorized by source (physician, patient, hospital, or not otherwise specified), type (education or experiences), disease site (cervical, endometrial, other), and user influence (number of posts, number of followers). Patient posts were specifically analyzed and all captions and hashtags were reviewed.

Results

The distribution of users with brachytherapy-related posts was as follows: 23% patients, 15% physicians, 9% hospitals, 53% not otherwise specified. Physicians only made up 11% of posts analyzed while the majority, 79%, were from patients and other Instagram users. From the accounts linked to patients, 99% of them were experience-based and 1% were educational. Posts made by physicians were educational in content 66% of the time, with 34% of posts being experiences. The median number of followers from least to greatest were not otherwise specified (NOS) 450.5, patients (501), hospital-affiliated (527), and physicians (608). In gynecological cancer patients, the reported side effects were as follows: fatigue 31%, gastrointestinal (GI) 16%, genitourinary (GU) 16%, pain 28%, and anxiety 50%.

Conclusion:

This study shows the influential power physicians have on social media and the need for increased brachytherapy awareness on platforms such as Instagram. Patients have voiced apprehension to pursue radiation due to lack of information provided and fear of the unknown. With this concern in mind, physicians are responsible to increase the availability of knowledge to patients in a more relaxed environment than the clinic. With increased physician social media presence, patients will have another avenue for support and reliable source of treatment information.

## Introduction

As social media continues to grow, it is vital to understand its impact on healthcare. Research on social media has increased and studies have investigated questions from public perception of disease to information dissemination [1]. Patients use social media to gather information and share experiences, which can influence patient decisions to accept therapy proposals raised by physicians.

Instagram is one of the image-based social media platforms with over one billion monthly active users [2]. Since it is widely available to the public, Instagram fosters the exchange of clinical information amongst multiple groups of people. As patients share their treatment journey, they form support groups and provide comfort to other patients suffering from the same disease through social media. Hashtags make it easier for patients to find other patients or healthcare professionals posting about their condition. Hashtags are phrases or words users include in their posts to associate their content with a specific topic. As more patients are treated with radiation for malignancies, their thoughts on brachytherapy treatment are shared on Instagram.

Brachytherapy uses sealed radioactive sources to deliver radiotherapy internally and has been used to treat multiple cancers including prostate, skin, sarcoma, breast, cervical and endometrial cancer. The vast majority of brachytherapy procedures in America are in locally advanced cervical cancer [3]. With the increasing use of brachytherapy in radiation oncology, we aimed to investigate the content and experiences shared by patients, medical professionals, and others on Instagram. This will guide us to improve patient care by communicating effectively using social media.

## Materials and methods

All posts on Instagram are publicly accessible. A list of top posts from searching #brachytherapy on May 7, 2021, were generated on a mobile device and all data are representative as of May 7, 2021. Searching for #brachytherapy resulted in 1010 posts which were analyzed using Instagram. The content was categorized by source (physician, patient, hospital, or not otherwise specified), type (education or experiences), disease site (cervical, endometrial, other), and user influence (number of posts, number of followers). Patient posts were specifically analyzed and all captions and hashtags were reviewed. Statistical analysis was run on the distribution of followers and posts among the user groups. The difference in the distribution of counts of followers across four groups was tested using the Kruskal-Wallis test. In the event that the global test was found to be significant, a pairwise comparison was done using the Wilcoxon sign rank test. 

## Results

A total of 1,010 Instagram posts were tagged with the hashtag brachytherapy and were analyzed for this study. 413 unique accounts were identified as either patient, MD, hospital, and not otherwise specified (NOS). The distribution of these accounts was as follows: 23% patients, 15% physicians, 9% hospitals, 53% not otherwise specified. (Table 1) 376 of the 1,010 posts analyzed were from accounts made by patients, 108 were from physicians, 101 were hospital affiliated and 425 were not otherwise specified.

When looking at the number of followers, physician accounts had the greatest number of followers. In order of greatest to least influential in terms of total followers were physicians, NOS, patients, and hospitals. The median number of followers from least to greatest were NOS (450.5), patients (501), hospital affiliated (527), and physicians (608). (Table 1)

**Table 1:** Analysis of Following Data SD: standard deviation

User	Number of accounts	Number of Followers	Mean	SD	Median
	Out of 413 linked				
Hospital Affiliated	36 (9%)	60,204	1672.33	3168.77	527
Physician	61 (15%)	231,788	3799.80	10857.92	608
Patients	96 (23%)	92,565	965.17	1483.71	501
Not otherwise specified	220 (53%)	182,949	1678.55	4654.74	450.
Difference in Distribution Of Followers (p-value)					
	Hospital Affiliated	Physician	Patients		
Physician	0.21				
Patients	0.90	0.04			
Not otherwise specified	0.84	0.01	0.84		

From the 96 accounts that were linked to patients, 99% of them were experience based and 1% were educational. Posts made by physicians were educational in content 66% of time, with 34% of posts being experiences. Out of the 36 accounts affiliated with hospitals, 86% were educational and 8% were experiences. (Table 2)

**Table 2:** Content of posts

	Educational	Experiences	Total number of posts
Patient	1%	99%	376
Physician	66%	34%	108
Hospital affiliated	86%	8%	425

An analysis was done comparing the distribution of the counts of followers and posts (separate analysis was done for each endpoint) across four study groups namely: hospital-affiliated, physician, patients, and others. The difference in the distribution of counts of followers across four groups was tested. The global test suggested a statistically significant difference across the four study groups for the counts of followers as well as for counts of posts. The pairwise comparison suggested a significant difference in the distribution of followers between physicians, and patients; between physician and other study groups. Similarly, there was a significant difference in the distribution of posts between physicians, and patients; between physician and other study groups; between patients and other study groups. A two-sided p-value of 0.05 was used to determine statistical significance. The analysis was done in R 4.0.1.

**Figure 1:**
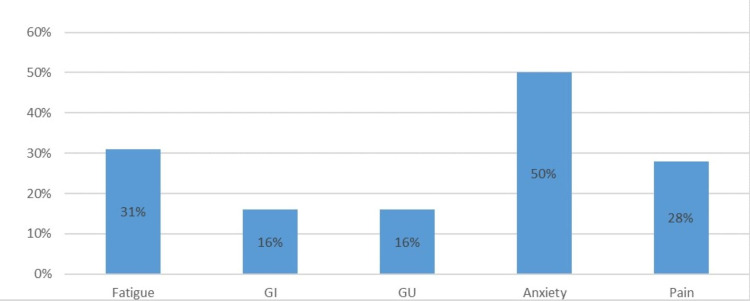
Gynecologic cancer patients' reported side effects on Instagram GI: Gastrointestinal, GU: Genitourinary

Out of 417 accounts, 65 patient accounts were linked to gynecological cancers. The #brachytherapy posts were analyzed for any side effects mentioned. The side effects were categorized by fatigue, gastrointestinal (GI), genitourinary (GU), pain, and anxiety. Out of the 65 accounts 32 of them mentioned side effects. 26 of these accounts were cervical cancer patients and six were endometrial. The reported side effects were as follows: fatigue 31%, GI 16%, GU 16%, pain 28%, and anxiety 50%. (Figure 1)

## Discussion

This study analyzes content relating to the presence of brachytherapy on Instagram. Physicians in Radiation Oncology have a large social media presence; however, the public is more likely to come across a post made by an unspecified source when searching for brachytherapy-related posts. Physicians only made up 11% of posts analyzed while the majority, 79%, were from patients and other Instagram users. We found that majority of brachytherapy posts on Instagram are from patients and their experiences. However, physician accounts have the greatest number of followers and are mostly educational in content. Physician accounts were educational only 66% of the time which is far less compared to a study showing 93% educational content posted by Dermatologists [4]. As the influence of social media continues to grow, an increasing number of patients will obtain medical information from these platforms. This suggests that Instagram can be used as an educational platform for patients.

This study showed 50% of patients analyzed with gynecological cancers reported anxiety. Brachytherapy is a different form of treatment that many patients are unaware of or are not accustomed to. A previous study showed the morbidity of significant psychological distress among 480 cervical cancer patients during radiotherapy was about 68% and stated the main factor causing emotional problems may be uncertainty about the disease [5]. Numerous patients voiced fear or anxiety in their posts because they were not knowledgeable on brachytherapy including diagnosis, treatment, and prognosis. Similarly, another study showed posttraumatic stress disorder (PTSD) in 41% of patients undergoing a specific brachytherapy procedure [6]. These psychological factors are important as they can cause increased cancer-specific mortality among patients with cervical cancer [7]. These results suggest a lack of information conveyed to patients on brachytherapy before they undergo treatment. Patients need additional education or counseling to reduce these unnecessarily high rates of anxiety.

Along with anxiety, fatigue was a major side effect reported by patients. Out of the posts analyzed, 31% of patients endorsed fatigue after or during treatment. A cross-sectional study looked at fatigue and quality of life in gynecological cancers found cancer-related fatigue was reported in 53% of women treated for gynecological cancers with a higher proportion in cervical cancer [8]. Fatigue worsens the quality of life for patients and can affect their ability to cope with the disease.

Previous data showed 20% of cervical cancer survivors have long-term bladder dysfunction [8]. Similarly, this study showed 16% of patients with gynecologic cancers reported GU symptoms. Although only #brachytherapy was analyzed, there are definitely more posts and hashtags related to brachytherapy that can be analyzed with future studies. Other limitations of this study include the lack of availability of demographic data made public on Instagram. Patient age, race, gender, and location were not publicly available.

As dissemination of information grows with social media usage, patients would benefit by posts increasing awareness of what brachytherapy entails in order to reduce their apprehensions. In addition, Instagram clearly provides a platform for patients to share experiences with one another in order to motivate and support each other. Physicians should be aware of the content posted on social media relating to their specialty and take advantage of this influential platform by finding ways to educate and support patients.

## Conclusions

This study suggests that radiation oncologists are influential on social media and there is a need for increased brachytherapy awareness on platforms such as Instagram. Physicians have the greatest number of followers compared with patients and other account holders and therefore can have a high level of impact on social media. Patients have voiced apprehension to pursue radiation due to lack of information provided and fear of the unknown. With this concern in mind, radiation oncologists along with other physicians are responsible to increase the availability of knowledge to patients in a more relaxed environment than the clinic. With increased physician social media presence, patients will have another avenue for support and reliable source of treatment information.
